# HRV and blood parameters for assessing the physiological functioning of cyclists during long-distance rides across different altitudes

**DOI:** 10.3389/fphys.2025.1559417

**Published:** 2025-06-17

**Authors:** Weiping Du, Ming Zhang, Xiaodan Niu, Hao Li, Yimin Wan

**Affiliations:** ^1^ School of Physical Education, Ningxia Normal University, Guyuan, Ningxia, China; ^2^ Center for Sports and Health Research, Ningxia Normal University, Guyuan, Ningxia, China; ^3^ Department of Public Basic Teaching Xi’an Academy of Fine Arts, Institute of Body-Medicine Integration, Xi’an, China; ^4^ School of Physical Education, Shaanxi Normal University, Xi’an, China; ^5^ School of Physical Education, Ningxia University, Yinchuan, Ningxia, China

**Keywords:** heart rate variability, blood parameters, autonomic nervous system, cycling athletes, high altitude

## Abstract

**Objective:**

This study aimed to systematically investigate the changes and interrelationships between heart rate variability (HRV) and hematological parameters in cyclists during prolonged exposure to varying altitudes, in order to reveal the dynamic interplay between autonomic nervous system regulation and hematological adaptation.

**Methods:**

Seventeen cycling enthusiasts aged 16–25 years participated in an 8-day altitude cycling challenge. HRV and hematological parameters were measured at three altitudes: 485 m, 1,627 m, and 4,182 m.

**Results:**

Hematological parameters, including white blood cell count (WBC), hemoglobin concentration (HGB), hematocrit (HCT), mean corpuscular hemoglobin concentration (MCHC), platelet count (PLT), and plateletcrit (PCT), significantly increased at both 1,627 m and 4,182 m (P < 0.05). Physiological measures such as heart rate (HR), systolic blood pressure (SBP), and diastolic blood pressure (DBP) showed significant elevations at 4,182 m (P < 0.05), while vital capacity (VC) significantly decreased (P < 0.05). HRV time-domain indices, including the standard deviation of R–R intervals (SDNN) and the root mean square of successive R–R interval differences (RMSSD), significantly increased at 1,627 m (P < 0.05) but decreased at 4,182 m (P < 0.05). Frequency-domain indices, including very low-frequency power (VLF), low-frequency power (LF), and high-frequency power (HF), significantly decreased at 4,182 m (P < 0.01). Among nonlinear HRV metrics, the short-term standard deviation of the Poincaré plot (SD1) and long-term standard deviation (SD2) significantly decreased at 4,182 m (P < 0.01), while approximate entropy (ApEn), sample entropy (SampEn), and alpha2 significantly increased (P < 0.05). Correlation analysis revealed that at 485 m, SDNN was negatively correlated with HCT (r = −0.55, P < 0.05) and PLT (r = −0.50, P < 0.05), while LF and HF were negatively correlated with HCT (r = −0.55 and −0.54, P < 0.05). At 1,627 m, SDNN was positively correlated with MCV (r = 0.53, P < 0.05), LF with MCV (r = 0.23, P < 0.05), and LF/HF was negatively correlated with MCHC (r = −0.52, P < 0.05). At 4,182 m, SDNN was positively correlated with MCHC (r = 0.51, P < 0.05), VLF was negatively correlated with WBC (r = −0.63, P < 0.05), ApEn was positively correlated with both WBC (r = 0.76, P < 0.05) and HCT (r = 0.62, P < 0.05), and SampEn was positively correlated with WBC (r = 0.74, P < 0.05).

**Conclusion:**

This study systematically evaluated the dynamic changes in HRV and hematological parameters in cyclists during prolonged exposure to different altitudes. The results showed that at moderate altitude, athletes exhibited a coordinated response of enhanced short-term autonomic adaptation and increased red blood cell volume. At very high altitude, HRV decreased overall while its complexity increased, indicating a stress-compensatory mechanism dominated by sympathetic activation. Altitude-specific correlations between HRV and blood parameters suggest a potential interplay between autonomic regulation and hematological adaptation.

## 1 Introduction

The Sichuan–Xizang Highway (G318), located in China, is a major national route connecting Chengdu and Lhasa, spanning a total length of 2,442 km. This highway traverses more than ten mountains with altitudes exceeding 4,000 m and passes through diverse natural landscapes such as forests, grasslands, glaciers, and snow-covered peaks. It is therefore known as China’s “Scenic Road.” At the same time, G318 is considered one of the most challenging and perilous roads in the world, attracting cycling enthusiasts from around the globe to test their limits.

In the study of extreme physical performance and feats of exceptional endurance, scientists have gradually uncovered the fundamental physiological characteristics of the human body. As early as 1925, Hill initiated investigations into human responses to extreme exercise, laying the foundation for our understanding of muscle fatigue (Hill, 1925). In endurance research, ultra-endurance exercise is commonly defined as running distances greater than 42.195 km or cycling over 100 miles (Hyldahl et al., 2024). Studies of such ultra-endurance efforts have revealed the body’s enormous demands for hydration and nutrition, along with subtle but significant changes in immune, metabolic, and endocrine functions. For example, Plasqui et al. (2019) assessed the energy expenditure of seven cyclists over a 24-day ride and found their average daily energy consumption reached 7,719 kcal. Similarly, Hyldahl et al. (2024) conducted a 17-day physiological assessment of a male cyclist and reported significant declines in vascular endothelial function and skeletal mitochondrial capacity.

Despite the growing body of research on ultra-endurance exercise, most studies have focused on performance outcomes and energy expenditure in elite athletes, with relatively limited attention paid to the assessment of cardiac autonomic nervous system regulation. Moreover, previous studies often involved small sample sizes, and some were limited to single-case reports. In addition, many studies have deliberately avoided high-altitude routes, leaving a gap in understanding how altitude-specific challenges influence physiological adaptation during cycling.

In prolonged, high-intensity cycling expeditions, athletes are subjected to multiple environmental stressors, among which altitude variation is a critical factor. Environmental physiology research conducted at high altitudes has shown that hypoxic conditions substantially alter the body’s exercise responses, primarily through mechanisms involving increased neuromuscular fatigue and compensatory activation of the sympathetic nervous system. As altitude increases, the decline in atmospheric oxygen partial pressure leads to reduced oxygen delivery to working muscles, which in turn accelerates the accumulation of metabolic byproducts and impairs mitochondrial function, thereby exacerbating peripheral fatigue (Ruggiero and McNeil, 2019). For lowland residents, acute high-altitude exposure can also induce central fatigue, characterized by reduced corticospinal excitability and impaired motor unit recruitment capacity (Ruggiero et al., 2022).

At the cellular level, adaptive responses such as plasma volume expansion and erythropoietin-induced erythropoiesis may partially counteract the limitations imposed by hypoxia on exercise capacity (Tymko et al., 2017). Furthermore, high-altitude exposure significantly activates the sympathetic nervous system, as evidenced by sustained increases in muscle sympathetic nerve activity (MSNA) and resetting of arterial baroreflex sensitivity (Simpson et al., 2019). In lowlanders, this sympathetic upregulation helps maintain arterial pressure but comes at the cost of compensatory tachycardia and potentially reduced vascular α-adrenergic receptor sensitivity (Simpson et al., 2019). These physiological adaptation mechanisms provide an important foundation for understanding individualized responses to exercise under hypoxic high-altitude conditions.

Building upon this research background, the present study conducted an 8-day cycling expedition along the G318 highway in China. A total of 17 participants (both male and female) rode an average of 8 h per day, covering over 700 km in total distance and accumulating more than 40 km in elevation gain. During this challenge, we selected three altitudes along the G318 route—485 m, 1,627 m, and 4,182 m—representing low, moderate, and very high altitude environments, respectively, to systematically investigate the physiological responses of the human body to different elevations.

Specifically, 485 m (Chengdu City) served as the low-altitude baseline, providing a reference point for evaluating subsequent high-altitude responses. The 1627-meter site (Xingou Town) represented a moderate-altitude transition zone, marking a critical phase where cyclists moved from normoxic to mildly hypoxic conditions, thereby allowing for the assessment of intermediate physiological adaptations. The 4182-meter site (Hani Town) reflected a very high altitude environment, where pronounced hypoxia is expected to significantly impact autonomic nervous function, metabolic processes, and energy balance (Siebenmann et al., 2024; Simpson et al., 2024). This gradient-based altitude design enabled us to systematically examine how incremental altitude gain affects physiological adaptation, autonomic regulation, and hematological responses in endurance cyclists. Moreover, the selected altitude points correspond to key checkpoints along the G318 route, ensuring ecological validity and realism in the field-based endurance challenge.

Therefore, this study aimed to systematically examine the changes and interrelationships between heart rate variability (HRV) and hematological parameters in cyclists during prolonged exposure to environments of varying altitudes—including low, moderate, and very high elevations. Considering the multidimensional nature of HRV as a marker of autonomic nervous system regulation, we employed a comprehensive analytical approach incorporating time-domain, frequency-domain, and nonlinear HRV indices. Specifically, time-domain measures (e.g., SDNN, RMSSD) were used to assess overall heart rate fluctuation and parasympathetic activity; frequency-domain parameters (e.g., VLF, LF, HF) enabled differentiation of sympathetic and parasympathetic spectral contributions; and nonlinear indices (e.g., ApEn, SampEn, SD1/SD2) captured the complexity of dynamic heart rate fluctuations, reflecting the body’s capacity for stress regulation in challenging environments. The combination of these three categories of HRV metrics allows for a more comprehensive characterization of cardiovascular-autonomic adaptation under the compound stressors of high-altitude exposure.

Based on current literature and known physiological mechanisms, we formulated the following hypotheses: (1) Exposure to moderate altitude (1,627 m) would lead to a temporary enhancement in HRV, indicating improved autonomic adaptability, while very high altitude (4,182 m) would result in a general decline across HRV indices, reflecting reduced autonomic regulation capacity; (2) HRV changes would exhibit significant correlations with hematological parameters (such as HGB, HCT, and WBC) at different altitudes, suggesting a coordinated interaction between autonomic regulation and hematological adaptation mechanisms.

## 2 Methods

### 2.1 Participants

Seventeen cycling enthusiasts aged 16–25 years participated in this study, including 12 males and 5 females, with a mean age of 21.18 years. All participants had extensive cycling experience and had successfully completed the Sichuan–Xizang Highway (G318) ride within the past year. Prior to the current challenge, all individuals underwent comprehensive medical examinations and were confirmed to be in good health with no known diseases. The study procedures and objectives were explained in detail to the participants, and informed consent was obtained prior to participation. All participants voluntarily joined the expedition. The study protocol was conducted in accordance with the Declaration of Helsinki and approved by the institutional review board of our organization (Approval Number: 20220901). Basic demographic and physical characteristics of the participants are presented in Table 1.

**TABLE 1:** Presents the basic information of the subjects.

Genders	Height (cm)	Weight (kg)	Age (years)	Basal metabolic rate (kcal)	HR (bpm)
Male (12)	176.00 ± 17.00	69.00 ± 8.05	21.58 ± 2.57	1832.02 ± 41.36	67.83 ± 5.51
Female (5)	162.00 ± 3.61	52.62 ± 3.97	20.20 ± 2.49	1,602.19 ± 24.01	77.00 ± 3.00

Cycling Plan: Based on logistical support conditions and the route of the Sichuan–Xizang Highway (G318), a strictly planned cycling schedule was implemented. Participants cycled along the G318 route, with detailed records of locations passed, daily riding distances, departure times, and cycling durations. These are summarized in Table 2.

**TABLE 2:** Schedule.

Date	Location	Departure time	Collect data time	Distance (km)	Average speed (km/h)	Slope (%)
Day 1	Chengdu City	8:30	7:30	154.00	17.11	0.64
Day 2	Ya’an City	8:30		88.00	11.00	3.48
Day 3	Xingou Town	8:30	7:30	115.00	12.78	6.32
Day 4	Zeduo Tang Town	8:30		59.00	8.43	7.61
Day 5	Xinduqiao Town	8:30		100.00	12.50	4.89
Day 6	Xiangkezong Town	8:30		81.00	8.53	4.59
Day 7	Honglong Town	8:30		96.00	12.00	4.05
Day 8	Hani Town		7:30	0.00	0.00	0.00

**FIGURE 1:**
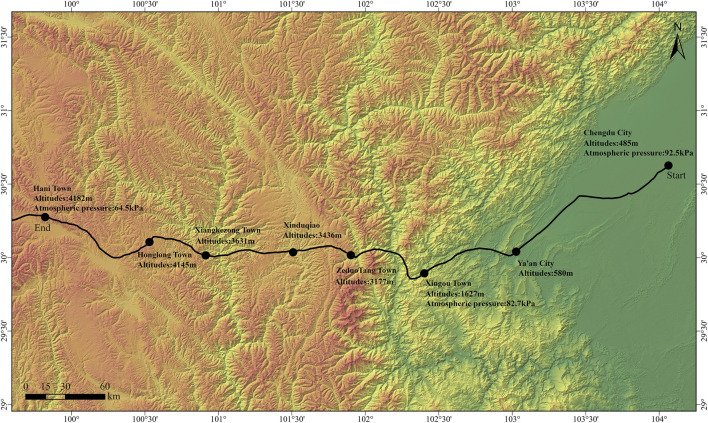
Cycling route information map.

Physiological parameters, hematological parameters, and HRV metrics were assessed at three different altitudes: Chengdu City (485 m), Xingou Town (1,627 m), and Hani Town (4,182 m). The specific information of the cycling route is shown in Figure 1.

### 2.2 Collection of hematological parameters and physiological parameters

Blood pressure: Systolic blood pressure (SBP) and diastolic blood pressure (DBP) were measured using a Yuwell YE680A upper arm blood pressure monitor (Jiangsu Yuwell Medical Equipment Co., Ltd., China).

Vital capacity (VC): Was assessed using a Jianmin FGC-A+ spirometer (Beijing Jianmin Medical Technology Co., Ltd., China), which employs a capacitance-based flow sensor. The device was recalibrated at each measurement site prior to data collection to account for local environmental conditions.

Hematological parameters: Under fasting conditions, 2 mL of venous blood was drawn from the antecubital vein using an ethylenediaminetetraacetic acid (EDTA) anticoagulant tube by qualified medical personnel. Hematological analyses, including white blood cell count (WBC), red blood cell count (RBC), hemoglobin concentration (HGB), hematocrit (HCT), mean corpuscular volume (MCV), mean corpuscular hemoglobin (MCH), mean corpuscular hemoglobin concentration (MCHC), platelet count (PLT), and plateletcrit (PCT), were performed using the Sysmex XS-500i automated hematology analyzer (Sysmex Corporation, Kobe, Japan), which was transported to each testing site and calibrated at least 30 min prior to sampling.

Given the potential influence of high-altitude barometric pressure on air-cushion aspiration systems, original manufacturer-sealed vacuum collection systems were used to ensure sample integrity. Additionally, the hematology analyzer underwent daily self-checks and quality control procedures at each altitude to ensure the accuracy and consistency of hematological measurements across different environmental conditions.

Data Quality Control Measures: To ensure the accuracy of the data and minimize fatigue-related confounding effects induced by daily cycling duration, the following procedures were implemented: Standardized rest and recovery: Participants adhered to a fixed rest schedule (Lights out at 10:30 p.m., wake-up at 7:10 a.m.) to minimize variability in recovery. Fasting measurements: All hematological samples were collected at 7:30 a.m. after an overnight fast to reduce acute effects from exercise or food intake. Consistent sampling time: HRV and hematological data were collected at the same time of day (early morning) at each altitude level to minimize the influence of circadian rhythm.

Energy intake: Daily dietary plans were designed by a certified nutritionist based on participants’ basal metabolic rate (BMR) and daily cycling duration. The average caloric intake ranged from 3,000 to 3,500 kcal/day, providing adequate energy to meet the physical demands of prolonged endurance exercise.

Supplementation: To eliminate potential confounding effects, the use of any nutritional supplements or antioxidant agents was strictly prohibited during the study period.

Hydration: Participants consumed an average of 3,500–4,500 mL of fluids per day, including bottled water and electrolyte-enriched beverages. Normal hydration status was ensured prior to all blood sampling and HRV assessments, with no signs of dehydration observed.

### 2.3 HRV data acquisition and analysis

Heart rate (HR) and RR interval data were collected using the BigRun BR-980 Pro wearable R-R interval recorder (Huitake Electronics Co., Ltd., Shenzhen, China), and HRV was analyzed using Kubios HRV Standard software (version 4.1.2; Kubios Oy, Kuopio, Finland).

Time-domain indices: Standard deviation of normal-to-normal R-R intervals (SDNN), root mean square of successive R-R interval differences (RMSSD), and the percentage of successive NN intervals differing by more than 50 ms (PNN50).

Frequency-domain indices: Very low-frequency power (VLF), low-frequency power (LF), high-frequency power (HF), and the ratio of LF to HF (LF/HF).

Nonlinear indices: Poincaré plot short-axis standard deviation (SD1) and long-axis standard deviation (SD2); approximate entropy (ApEn), sample entropy (SampEn); and detrended fluctuation analysis scaling exponents (Alpha1 and Alpha2).

RR interval data were recorded from 7:30 to 8:00 a.m. on the morning following arrival at each altitude site. Participants were instructed to lie in a supine position with eyes closed, and data were collected continuously for 10 min. The sampling rate was set to 1,000 Hz.

In addition, in accordance with the 2024 version of the *Kubios HRV User Guide* and previous literature (Tarvainen et al., 2014), we performed preprocessing of RR interval data in the Kubios software to ensure more accurate HRV analysis. The specific procedures were as follows:

(1) Artifact detection and correction: The built-in automatic artifact detection function in Kubios was used to identify abnormal RR intervals based on the default algorithm. All detected artifacts were corrected using cubic spline interpolation, aiming to minimize the impact of non-physiological disturbances on HRV metrics.
(2) Resampling and interpolation: To meet the requirement for equally spaced time series in frequency-domain and nonlinear analyses, RR intervals were interpolated using a cubic spline at a resampling rate of 4 Hz. This approach is in line with Kubios recommendations and widely adopted in short-term HRV analysis.
(3) Detrending: Low-frequency trends were removed using the Smoothness Priors method with a default Lambda value of 500. This preprocessing step helps eliminate slow drifts caused by posture changes or device shifts, thereby improving the stability of HRV time series.

### 2.4 Data statistics

All data analyses and visualizations were performed using Microsoft Excel (Version 2019; Microsoft Corporation, Redmond, Washington, United States), SPSS (Version 24.0; SPSS Inc., Chicago, IL, United States), and GraphPad Prism (Version 10.1.2; GraphPad Software, San Diego, CA, United States). The Kolmogorov–Smirnov test was used to assess the normality of data distributions, and Levene’s test was applied to evaluate homogeneity of variances.

For physiological and hematological parameters that met the assumptions of normality and homogeneity of variance, repeated measures analysis of variance (repeated measures ANOVA) was conducted to assess changes across different altitude conditions. When a significant main effect was observed, Bonferroni-adjusted pairwise comparisons were performed for *post hoc* analysis to control the risk of type I error associated with multiple comparisons. For HRV variables that did not satisfy normality or homogeneity of variance assumptions, the Friedman test was used as a non-parametric alternative. Post hoc comparisons were conducted using the Dunn–Bonferroni method. In addition, Spearman correlation analyses were performed to examine the relationships between HRV and hematological parameters under different altitude conditions. All data are reported as median [25th–75th percentile]. P < 0.05 was considered statistically significant.

## 3 Results

### 3.1 Hematological parameters and physiological parameters

**FIGURE 2:**
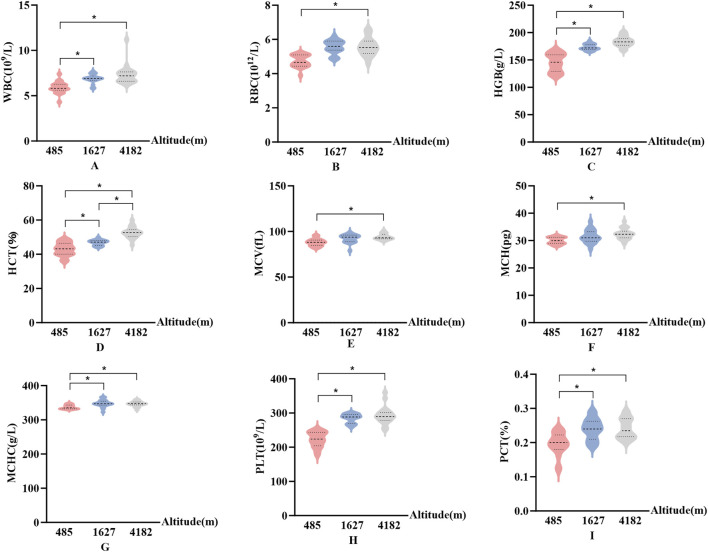
Changes in hematological parameters at different altitudes **(A)**: White blood cell count (WBC); **(B)** Red blood cell count (RBC); **(C)** Hemoglobin concentration (HGB); **(D)** Hematocrit (HCT); **(E)** Mean corpuscular volume (MCV); **(F)** Mean corpuscular hemoglobin (MCH); **(G)** Mean corpuscular hemoglobin concentration (MCHC); **(H)** Platelet count (PLT); **(I)** Plateletcrit (PCT); vs. 485 m or 1627 m, *: P < 0.05).

In Figure 2, Significant altitude-related differences were observed in several hematological indices, including WBC, HGB, MCHC, PLT, and PCT. These parameters showed consistent upward trends. Specifically, compared to 485 m, values at both 1,627 m and 4,182 m were significantly elevated (P < 0.05, F > 9.9). No significant changes were observed for RBC, MCV, or MCH at 1,627 m. However, at 4,182 m, both MCV and MCH were significantly increased compared to 485 m (P < 0.05, F > 1.3). Although RBC showed a decreasing trend from 1,627 m to 4,182 m, this change was not statistically significant. Additionally, HCT exhibited a significant altitude-dependent increase. HCT values were significantly higher at both 1,627 m and 4,182 m compared to 485 m (P < 0.05), and further increased from 1,627 m to 4,182 m (P < 0.05, F > 1.4).

**FIGURE 3:**
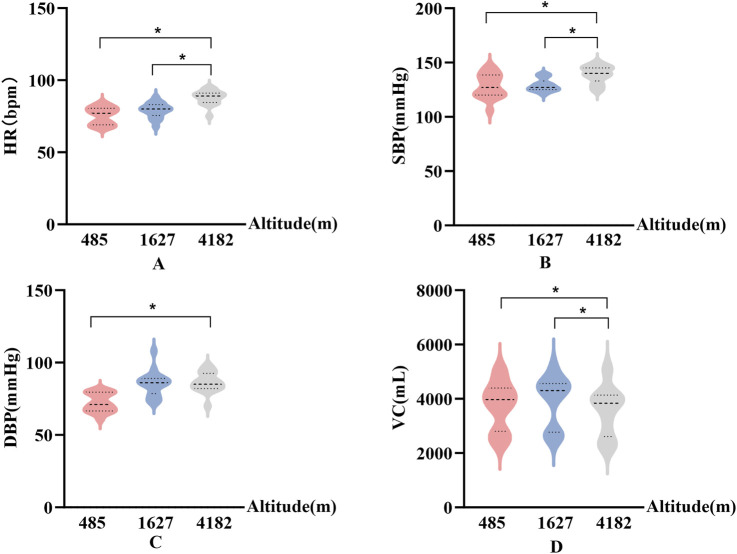
Changes in physiological parameters at different altitudes **(A)**: Heart rate (HR); **(B)** Systolic blood pressure (SBP); **(C)** Diastolic blood pressure (DBP); **(D)** Vital capacity (VC); vs. 485 m or 1627 m, *: P < 0.05).

In Figure 3, HR, SBP, DBP, and VC exhibited significant differences across altitude levels. Specifically, compared to 485 m, both HR and SBP were significantly elevated at 4,182 m (P < 0.05). In addition, SBP showed a further significant increase from 1,627 m to 4,182 m (P < 0.05, F > 12.1).

VC decreased significantly at 4,182 m compared to both 485 m and 1,627 m (P < 0.05, F = 18). DBP was significantly higher at 4,182 m than at 485 m (P < 0.05). Notably, SBP demonstrated a greater magnitude of change in response to high-altitude exposure compared to DBP.

### 3.2 HRV results

**FIGURE 4:**
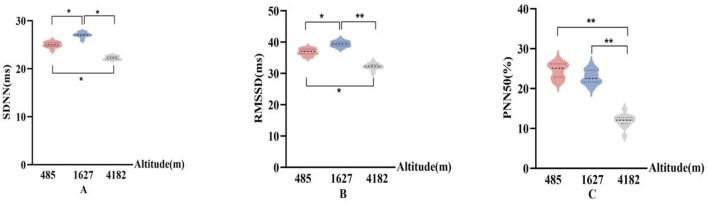
Changes in time-domain HRV parameters at different altitudes **(A)**: Standard deviation of R–R intervals (SDNN); **(B)** Root mean square of successive R–R interval differences (RMSSD); **(C)** Percentage of successive normal sinus intervals differing by more than 50 ms (PNN50); vs. 485 m or 1627 m, *: P < 0.05; **: P < 0.01).

In Figure 4, SDNN and RMSSD showed consistent patterns across different altitude levels. Compared to 485 m, both SDNN and RMSSD significantly increased at 1,627 m (P < 0.05), indicating a temporary enhancement in HRV. However, at 4,182 m, SDNN and RMSSD values significantly decreased compared to both 485 m and 1,627 m (P < 0.05 or P < 0.01). PNN50 did not show significant changes at 1,627 m. However, PNN50 demonstrated a significant downward trend at 4,182 m compared to both 485 m and 1,627 m (P < 0.01), suggesting impaired parasympathetic modulation under very high altitude conditions.

**FIGURE 5:**
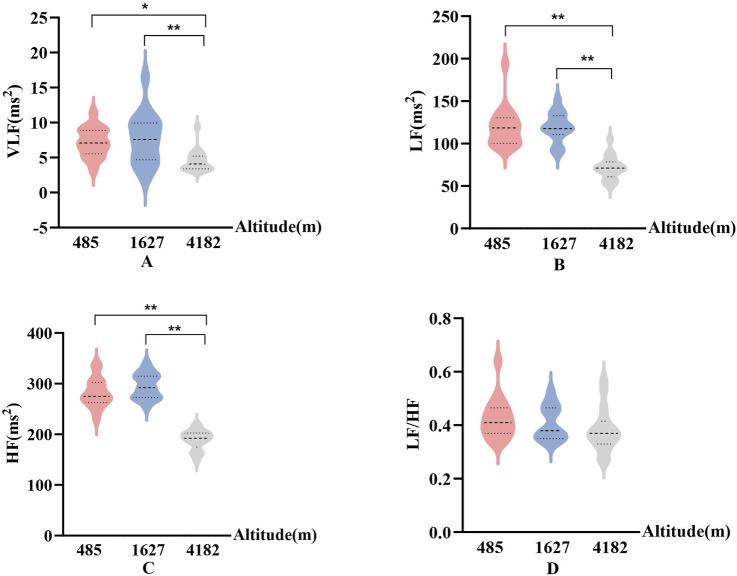
Frequency-domain HRV parameters at different altitudes (**(A)**: Very low-frequency power (VLF); **(B)** Low-frequency power (LF); **(C)** High-frequency power (HF); **(D)** The ratio of LF to HF (LF/HF); vs. 485 m or 1627 m, *: P < 0.05; **: P < 0.01).

**FIGURE 6:**
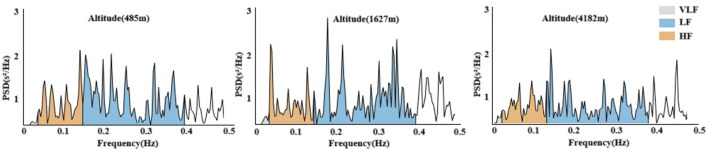
Power spectral density changes in HRV across altitudes.

In Figures 5, 6, Significant changes were observed in the frequency-domain HRV parameters. At 1,627 m, VLF, LF, and HF showed notable variations. At 4,182 m, VLF, LF, and HF values demonstrated significant decreases compared to both 485 m and 1,627 m (P < 0.05 or P < 0.01). However, the LF/HF ratio remained statistically unchanged throughout the entire cycling period. These results suggest that HRV frequency-domain indices are more susceptible to high-altitude influence, particularly under very high altitude conditions.

**FIGURE 7:**
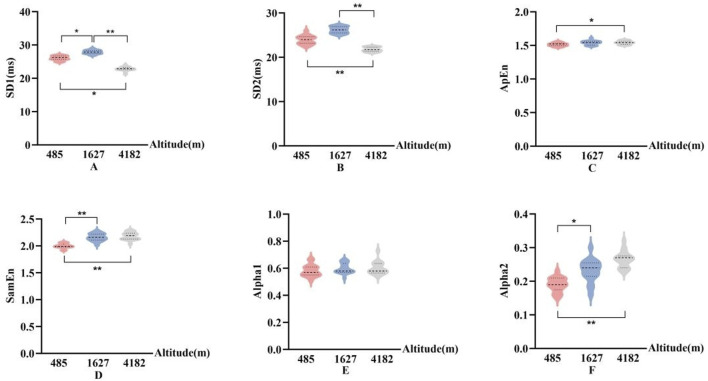
Changes in nonlinear HRV parameters at different altitudes (**(A)**: Poincaré plot short-axis standard deviation (SD1); **(B)**:Poincaré plot long-axis standard deviation (SD2); **(C)**: Approximate entropy (ApEn); **(D)**: sample entropy (SampEn); **(E)**: Alpha1; **(F)**: Alpha2; vs. 485 m or 1627 m, *: P < 0.05; **: P < 0.01).

**FIGURE 8:**
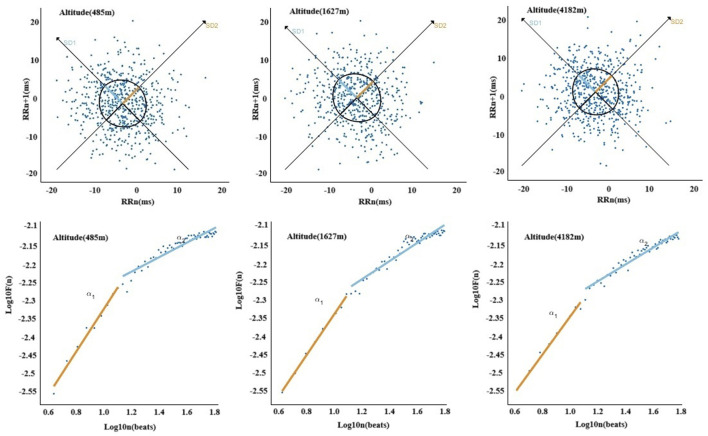
Altitude-related changes in Poincaré plot and detrended fluctuation analysis.

In Figures 7, 8, The nonlinear HRV parameters exhibited more complex patterns across different altitudes. Compared to 485 m, at 1,627 m, there were significant increases in SD1), ApEn, and Alpha2 (P < 0.05 or P < 0.01). At 4,182 m, both SD1 and the long-axis standard deviation (SD2) significantly decreased (P < 0.01), whereas ApEn, SampEn, and Alpha2 significantly increased (P < 0.05 or P < 0.01). No significant changes in SD2 or ApEn were observed at 1,627 m. Furthermore, when comparing 4,182 m–1,627 m, both SD1 and SD2 showed significant declines (P < 0.01).

### 3.3 Correlation results

**FIGURE 9:**
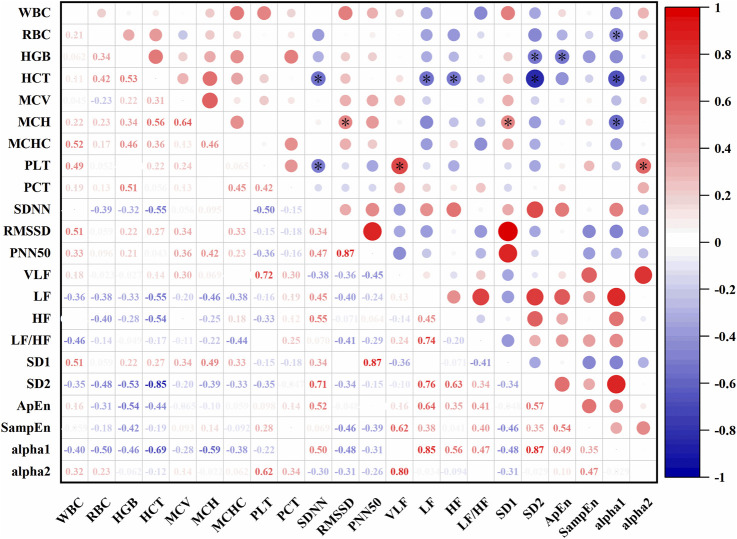
Correlations between HRV and hematological parameters at 485 m (*: P < 0.05).

In Figure 9, At an altitude of 485 m, several significant correlations were observed between HRV and hematological parameters. SDNN was negatively correlated with HCT (r = −0.55, P < 0.05) and PLT (r = −0.50, P < 0.05). VLF was positively correlated with PLT (r = 0.72, P < 0.05). LF showed a negative correlation with HCT (r = −0.55, P < 0.05), and HF was also negatively correlated with HCT (r = −0.54, P < 0.05). SD1 was positively correlated with MCH (r = 0.49, P < 0.05), while SD2 was negatively correlated with HCT (r = −0.85, P < 0.05) and HGB (r = −0.55, P < 0.05). ApEn showed a negative correlation with HGB (r = −0.54, P < 0.05). Alpha1 was negatively correlated with MCH (r = −0.59, P < 0.05), HCT (r = −0.69, P < 0.05), and RBC (r = −0.50, P < 0.05). Alpha2 was positively correlated with PCT (r = 0.62, P < 0.05).

**FIGURE 10:**
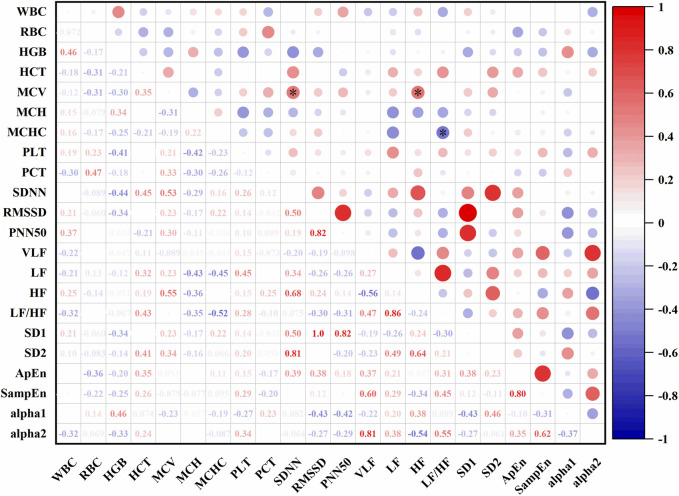
Correlations between HRV and hematological parameters at 1,627 m (*: P < 0.05).

In Figure 10, At an altitude of 1,627 m, SDNN was positively correlated with mean corpuscular volume (MCV) (r = 0.53, P < 0.05), LF was positively correlated with MCV (r = 0.23, P < 0.05), and LF/HF was negatively correlated with MCHC (r = −0.52, P < 0.05).

**FIGURE 11:**
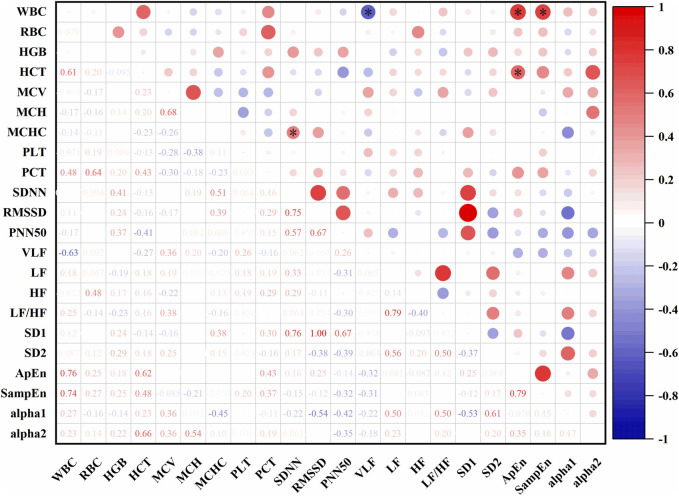
Correlations between HRV and hematological parameters at 4,182 m (*: P < 0.05).

In Figure 11, At an altitude of 4,182 m, SDNN was positively correlated with MCHC (r = 0.51, P < 0.05). VLF was negatively correlated with white blood cell count (WBC) (r = −0.63, P < 0.05). ApEn showed positive correlations with both WBC (r = 0.76, P < 0.05) and HCT (r = 0.62, P < 0.05). SampEn was also positively correlated with WBC (r = 0.74, P < 0.05).

## 4 Discussion

### 4.1 Effects of altitude on hematological parameters and physiological parameters

This study found that upon reaching altitudes of 1,627 m and 4,182 m, athletes exhibited significant increases in hemoglobin concentration (HGB), mean corpuscular hemoglobin concentration (MCHC), platelet count (PLT), and plateletcrit (PCT). Previous research has shown that, during initial high-altitude exposure in lowland residents, a rapid reduction in plasma volume (PV) is the primary mechanism driving increased hemoglobin concentration. Over time, expansion of red cell volume (RCV) further contributes to hemoconcentration (Siebenmann et al., 2024; Płoszczyca et al., 2018; Lobigs et al., 2017). These mechanisms are highly consistent with the observed increases in HGB, MCHC, PLT, and PCT in the current study, suggesting that the athletes underwent a similar pattern of hematological adaptation. Although red blood cell count (RBC), mean corpuscular volume (MCV), and mean corpuscular hemoglobin (MCH) did not significantly change at 1,627 m, all three indices showed significant alterations at 4,182 m. This suggests that physiological adaptations in hematological profiles may become more pronounced at very high altitudes (Wang et al., 2018; Wang et al., 2022). In addition, hematocrit (HCT) increased progressively with altitude and was significantly elevated at both 1,627 m and 4,182 m compared to 485 m. It has been reported that reductions in total circulating protein (TCP) at high altitude may lead to changes in colloid osmotic pressure, promoting fluid shifts from the intravascular to interstitial compartments. This mechanism accelerates plasma volume loss and contributes to hemoconcentration (Okazaki et al., 2019; Young et al., 2019). These findings may help explain the sustained rise in HCT observed in this study, particularly at very high altitudes. The observed increase in white blood cell count (WBC) may be associated with a nonspecific immune response triggered by environmental stress (Basak et al., 2021). Swenson et al. (2020) also reported that high-altitude exposure can lead to shedding of the endothelial glycocalyx, which subsequently promotes plasma protein extravasation and immune activation. These findings are consistent with the elevated WBC levels observed at altitudes of 1,627 m and 4,182 m in the present study. Such responses may not only affect athletic performance but could also have significant implications for training and competition strategies in high-altitude environments (Nummela et al., 2020).

In terms of physiological responses, this study found that cycling across high-altitude environments led to significant changes in heart rate (HR), systolic blood pressure (SBP), diastolic blood pressure (DBP), and vital capacity (VC). Specifically, all three cardiovascular parameters—HR, SBP, and DBP—were significantly elevated at 4,182 m, while VC was significantly reduced. These changes are likely associated with autonomic nervous system adjustments in response to altitude-induced hypoxia. However, it is important to note that reduced air density at high altitude may interfere with flow sensor performance, potentially affecting the accuracy of VC measurements. Future studies are encouraged to adopt spirometry systems specifically designed for high-altitude environments to ensure greater measurement reliability. HR and blood pressure alterations are well-recognized physiological responses to high-altitude exposure. Previous studies have shown that acute exposure to high altitude triggers autonomic regulation of cardiovascular function to enhance oxygen delivery and utilization efficiency (Vivek et al., 2018; Kicken et al., 2019; Stembridge et al., 2018). In this study, the significant increases in HR and SBP at 4,182 m likely reflect a stress-induced compensatory response aimed at maintaining adequate tissue oxygenation under hypoxic conditions. The concurrent rise in DBP further supports this interpretation, indicating an increased cardiovascular load during very high altitude exposure (Zhang et al., 2022).

In this study, a significant decrease in vital capacity (VC) was observed at an altitude of 4,182 m, which may result from a combination of interacting physiological mechanisms. Previous research has shown that high-altitude hypoxia can induce pulmonary vasoconstriction, leading to elevated pulmonary arterial pressure and increased permeability of the alveolar-capillary membrane. These changes facilitate fluid leakage into the alveolar space, contributing to the development of diffuse subclinical pulmonary edema (Walker et al., 2021; Mazur et al., 2020; Kurtzman and Caruso, 2018). In addition, prolonged endurance exercise combined with hypoxic stress may lead to respiratory muscle fatigue, particularly affecting the diaphragm and accessory inspiratory muscles, thereby limiting thoracic expansion and ventilatory capacity. Caspersen et al. (2013) further noted that cold temperatures and hypoxia at high altitude can provoke bronchoconstriction, increasing expiratory resistance and exacerbating airway limitation, which collectively impair pulmonary function. Therefore, the observed reduction in VC during cycling at very high altitude is likely attributable to multiple high-altitude adaptive responses, including subclinical pulmonary edema, respiratory muscle fatigue, and bronchoconstriction.

### 4.2 Effects of altitude on time-domain HRV indices

This study found that when athletes ascended from 485 m to 1,627 m (moderate altitude), both the standard deviation of R–R intervals (SDNN) and the root mean square of successive R–R interval differences (RMSSD) increased significantly, indicating a short-term improvement in heart rate variability (HRV). Simpson et al. (2024) reported that during early acute hypoxic exposure, the enhancement of muscle sympathetic nerve activity (MSNA) is primarily characterized by an increase in burst frequency, while burst incidence remains unchanged. This pattern suggests that the increase in heart rate passively elevates the number of sympathetic bursts per unit time, without a corresponding rise in central sympathetic drive. Such a relatively mild sympathetic activation may allow the parasympathetic nervous system to remain functionally active, thereby contributing to the transient elevation in HRV parameters such as SDNN and RMSSD at moderate altitude. It is important to note, however, that the observed improvement in HRV at 1,627 m should not be interpreted as a sign of enhanced cardiac health. According to Stanley et al. (2013), such transient increases in HRV are more likely to reflect short-term autonomic adaptations to acute physiological stressors (e.g., hypoxia or exercise), rather than long-term improvements in cardiac function. This temporary enhancement in parasympathetic activity may help stabilize cardiovascular regulation during early exposure but requires further investigation to determine its long-term significance.

In contrast, upon reaching 4,182 m, both SDNN and RMSSD significantly declined, accompanied by a marked reduction in PNN50. Previous studies have shown that increases in SDNN are typically associated with better cardiac adaptability, while RMSSD is more reflective of parasympathetic activity (Siaplaouras et al., 2021; Choo et al., 2024). The findings of the present study suggest that at very high altitude, the autonomic nervous system’s adaptive capacity is diminished, with elevated sympathetic dominance and suppressed parasympathetic activity leading to an overall reduction in HRV. This aligns with earlier research indicating that high-altitude hypoxic exposure attenuates cardiovascular autonomic regulation (Bhattarai et al., 2018).

### 4.3 Effects of altitude on frequency-domain HRV indices

In this study, frequency-domain HRV indices showed significant decreases in very low-frequency power (VLF), low-frequency power (LF), and high-frequency power (HF) at 4,182 m. Previous research suggests that altitude-induced increases in sympathetic activity may suppress parasympathetic modulation, reflected by a decrease in HF. Additionally, hypoxia may directly inhibit low-frequency oscillations via central mechanisms, such as activation of rostral ventrolateral medulla (RVLM) neurons (Oshima et al., 2018; Herzig, 2024). These mechanisms are consistent with the observed simultaneous decline in VLF, LF, and HF in the present study. Changes in exercise intensity are also known to influence frequency-domain HRV parameters. After high-intensity exertion, it is common to observe a decrease in HF and an increase in LF (Waghmare et al., 2024). Although no significant changes in HRV frequency components were detected at moderate altitude in this study, the marked reductions at very high altitude may be attributed to both increased physical exertion during prolonged cycling and reduced oxygen availability. Interestingly, the LF/HF ratio remained unchanged throughout the cycling period. This may indicate a relatively stable balance between sympathetic and parasympathetic modulation across altitudes. However, some studies argue that a stable LF/HF ratio—despite simultaneous reductions in both LF and HF—may actually reflect concurrent suppression of both autonomic branches rather than preserved autonomic balance (Simpson et al., 2024; Tanoue et al., 2021). This interpretation offers a novel perspective on the results observed in this study.

### 4.4 Effects of altitude on nonlinear HRV indices

In the context of nonlinear heart rate variability (HRV) analysis, SD1 reflects short-term variability of R–R intervals and is primarily associated with instantaneous parasympathetic modulation. SD2 represents long-term variability and is influenced by both sympathetic and parasympathetic nervous system activity (Tarvainen et al., 2014). Approximate entropy (ApEn) and sample entropy (SampEn) are commonly used to assess the complexity and irregularity of R–R interval time series; higher values typically indicate enhanced diversity and adaptability of autonomic regulation (Bolea et al., 2016). Alpha1 and Alpha2 quantify fractal scaling properties over short and long time scales, respectively, with elevated Alpha2 often interpreted as a marker of increased sympathetic activity and altered long-term autonomic regulation (Tarvainen et al., 2014).

In this study, SD1, SampEn, and Alpha2 significantly increased at 1,627 m, suggesting a mixed autonomic adaptive response under moderate altitude exposure. The rise in SD1 may reflect transient enhancement of parasympathetic activity, while the elevation in SampEn indicates increased heart rate complexity. The concurrent increase in Alpha2 may represent early signs of sympathetic activation. This combination—enhanced parasympathetic tone, increased complexity, and rising Alpha2—suggests that at moderate altitude, the autonomic nervous system retains flexibility and dynamic balance, allowing the body to effectively cope with mild hypoxia and physical exertion.

However, upon reaching 4,182 m, both SD1 and SD2 decreased significantly, indicating reduced magnitude and flexibility of autonomic modulation. At the same time, ApEn, SampEn, and Alpha2 continued to rise. This pattern suggests that the increase in HRV complexity at very high altitude may no longer reflect adaptive regulation but rather a shift toward a sympathetically dominant and potentially dysregulated state. This interpretation aligns with the mechanisms proposed by Simpson et al. (2024), who suggested that severe hypoxia, elevated pulmonary arterial pressure, and central nervous system inflammation may enhance respiratory-sympathetic coupling, thereby altering nonlinear HRV characteristics. Boos et al. (2022) also reported that in states of intense or prolonged physiological stress, elevated HRV complexity indices may coincide with autonomic imbalance rather than improved regulation.

Therefore, the nonlinear HRV responses observed in this study can be interpreted as a progression from short-term adaptive regulation to a state of sympathetic-dominated physiological stress. Notably, under conditions of very high altitude, the increase in HRV complexity may reflect cumulative regulatory strain and potential functional instability, rather than a continuation of healthy adaptation. These findings underscore the value of nonlinear HRV indices in capturing dynamic shifts in autonomic regulation under extreme environmental stress.

### 4.5 Correlation between HRV and hematological parameters at different altitudes

By integrating time-domain, frequency-domain, and nonlinear HRV metrics with hematological parameters, this study systematically revealed the dynamic interplay between autonomic regulation and hematological adaptation under varying altitude conditions. Specifically, in the low-altitude environment (485 m), HRV indices such as SDNN, LF, and HF were negatively correlated with hemoconcentration-related parameters like hematocrit (HCT) and platelet count (PLT). In particular, the negative correlation between SDNN and HCT (r = −0.55) suggests that under well-oxygenated conditions, increased blood viscosity may be associated with reduced autonomic nervous activity. Previous studies have shown that elevated red cell volume may increase blood viscosity, raising cardiac afterload and consequently reducing HRV (Castilho et al., 2018; Cooper et al., 2015).

At moderate altitude (1,627 m), both SDNN and LF were positively correlated with mean corpuscular volume (MCV), indicating that in mildly hypoxic conditions, an increase in MCV may be accompanied by enhanced autonomic modulation. This coordinated increase in blood viscosity and HRV likely reflects a short-term adaptive mechanism in which the cardiovascular system adjusts dynamically to optimize oxygen delivery (Yperzeele et al., 2016; Barutcu et al., 2011). At very high altitude (4,182 m), the pattern of correlations shifted markedly. SDNN was positively correlated with mean corpuscular hemoglobin concentration (MCHC), VLF was negatively correlated with white blood cell count (WBC), and ApEn showed positive correlations with both WBC and HCT. These findings suggest that under extreme hypoxia and heightened systemic inflammation, traditional amplitude-based HRV indices such as SDNN tend to decrease, while nonlinear complexity measures such as ApEn and SampEn increase. This pattern may reflect a compensatory response in which the body attempts to preserve physiological stability by enhancing heart rate complexity in the face of impaired autonomic control (Bosco et al., 2016; Hildenborg et al., 2022).

## 5 Limitations and future directions

Although this study provides preliminary insights into the physiological and autonomic responses of athletes during prolonged cycling across different altitudes, several limitations should be acknowledged. First, data were collected at only three discrete altitude levels without continuous or periodic monitoring throughout the entire route. Additionally, there were unequal intervals between measurement points, which may introduce bias. This design was primarily constrained by the actual geographical and logistical conditions of the cycling route. The selection of three representative altitudes—low, moderate, and very high—was based on key elevation milestones along the G318 highway and determined through a combination of factors including logistical feasibility, safety, accessibility, and recovery conditions for participants. Nonetheless, we acknowledge that the asymmetry in sampling intervals and cumulative exercise load may have influenced the hematological and HRV outcomes. For example, the final measurement at 4,182 m may reflect not only altitude-related stress but also accumulated fatigue or adaptive responses, potentially confounding the interpretation of autonomic and hematological changes. Future studies should aim to refine the temporal symmetry of data collection and incorporate training load quantification tools such as the Training Impulse (TRIMP) score. Additionally, including a control group or implementing a crossover design could help disentangle the effects of altitude acclimatization from those of physical fatigue or recovery, enabling a more precise understanding of their independent contributions to physiological regulation during high-altitude endurance exercise.

Secondly, this correlational analysis was intended as an exploratory step to identify potential associations between hematological parameters and heart rate variability (HRV) indices. We acknowledge that no correction for multiple comparisons was applied, which may increase the risk of Type I error. Future studies with larger sample sizes should incorporate appropriate multiple testing corrections to validate these preliminary findings.

## 6 Conclusion

This study systematically evaluated the dynamic changes in HRV and hematological parameters in cyclists during prolonged exposure to different altitudes. The results showed that at moderate altitude, athletes exhibited a coordinated response of enhanced short-term autonomic adaptation and increased red blood cell volume. At very high altitude, HRV decreased overall while its complexity increased, indicating a stress-compensatory mechanism dominated by sympathetic activation. Altitude-specific correlations between HRV and blood parameters suggest a potential interplay between autonomic regulation and hematological adaptation. This study offers new insights into physiological adaptation pathways during endurance exercise at high altitude.

## Data Availability

The raw data supporting the conclusions of this article will be made available by the authors, without undue reservation.
